# Symptomatic knee disorders in floor layers and graphic designers. A cross-sectional study

**DOI:** 10.1186/1471-2474-13-188

**Published:** 2012-09-25

**Authors:** Lilli Kirkeskov Jensen, Søren Rytter, Jens Peter Bonde

**Affiliations:** 1 Department of Occupational and Environmental Medicine, Bispebjerg Hospital, Bispebjerg Bakke 23, Copenhagen, DK 2400, NV, Denmark; 2 Department of Orthopaedic Surgery, Hospital Unit West, Lægårdvej 12, Holstebro, DK 7500, Denmark

**Keywords:** Epidemiology, Occupational, Knee, Osteoarthritis, KOOS

## Abstract

**Background:**

Previous studies have described an increased risk of developing tibio-femoral osteoarthritis (TF OA), meniscal tears and bursitis among those with a trade as floor layers. The purpose of this study was to analyse symptomatic knee disorders among floor layers that were highly exposed to kneeling work tasks compared to graphic designers without knee-demanding work tasks.

**Methods:**

Data on the Knee injury and Osteoarthritis Outcome Score (KOOS) were collected by questionnaires. In total 134 floor layers and 120 graphic designers had a bilateral radiographic knee examination to detect TF OA and patella-femoral (PF) OA. A random sample of 92 floor layers and 49 graphic designers had Magnetic Resonance Imaging (MRI) of both knees to examine meniscal tears. Means of the subscales of KOOS were compared by analysis of variance. The risk ratio of symptomatic knee disorders defined as a combination of radiological detected knee OA or MRI-detected meniscal tears combined with a low KOOS score was estimated by logistic regression in floor layers with 95% confidence interval (CI) and adjusted for age, body mass index, traumas, and knee-straining sports activities. Symptomatic knee OA or meniscal tears were defined as a combination of low KOOS-scores and radiographic or MRI pathology.

**Results:**

Symptomatic TF and medial meniscal tears were found in floor layers compared to graphic designers with odds ratios 2.6 (95%CI 0.99-6.9) and 2.04 (95% CI 0.77-5.5), respectively. There were no differences in PF OA. Floor layers scored significantly lower on all KOOS subscales compared to graphic designers. Significantly lower scores on the KOOS subscales were also found for radiographic TF and PF OA regardless of trade but not for meniscal tears.

**Conclusions:**

The study showed an overall increased risk of developing symptomatic TF OA in a group of floor layers with a substantial amount of kneeling work positions. Prevention would be appropriate to reduce the proportion of kneeling postures e.g. by working with tools used from a standing working position.

## Background

Previous studies described an increased risk of developing tibio-femoral osteoarthritis (TF OA) and meniscal tears among floor layers with high knee loads compared to referents without knee straining work [1-6]. Danish floor layers have the same job functions when installing floorings of carpets, linoleum, vinyl, and rubber - and kneeling positions constitute a substantial proportion of their work day. Previous studies have shown that there is not necessarily a correlation between radiological findings and clinical symptoms including knee pain [7-9]. Radiological knee OA and other para-clinical and clinical findings are only clinically relevant if associated with symptoms or disability.

The Knee injury and Osteoarthritis Outcome Score (KOOS) was developed as an instrument to assess knee symptoms and disability. KOOS has been validated [10] and has been used in several clinical studies involving patients with meniscal injuries and knee OA. It has been used to evaluate effects of treatment [10]. No studies have been found using the score to ascertain work-related symptomatic knee disorders.

The purpose of this study was to examine the risk of symptomatic knee disorders among floor layers who experience a high amount of kneeling work tasks compared to graphic designers without knee-demanding work tasks. Symptomatic knee disorders are defined here as a combination of radiographically detected knee OA or Magnetic Resonance Imaging (MRI)-detected meniscal tears combined with a low KOOS score. In addition, we investigated the general association of KOOS subscales with radiographically and MRI-detected OA and meniscal tears, independent of trade.

## Methods

### Study sample

A cohort of 286 Danish floor layers and 370 graphic designers was established in 1994 based on membership lists from trade unions. All males, aged 36–70 years in 2004, still employed and those who had have left the trade were included in this study. Danish floor layers and graphic designers are comparable with respect to level of education and socio-economic status. Questionnaires were mailed to floor layers and graphic designers in 2004–2005, and the response rates were 89 and 78%, respectively. Information on age, height, weight, years in the trade, knee complaints (Nordic Health Questionnaire and the KOOS) knee traumas, and knee-straining sports activity were collected by questionnaires.

**Figure 1:**
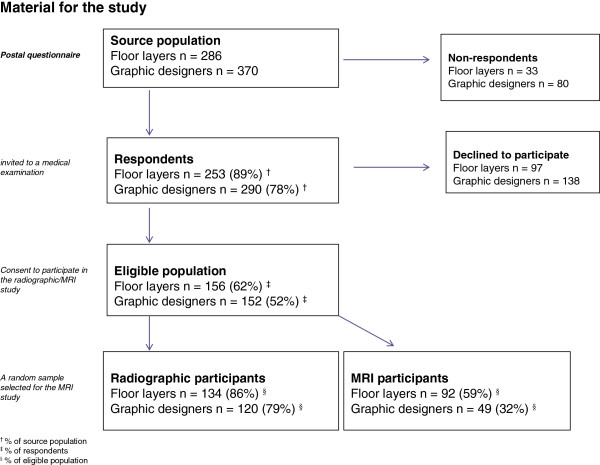
Material for the study.

Questionnaire respondents were invited to participate in clinical and radiographic examinations. Informed consent to participate was given by 156 floor layers and 152 graphic designers. Consent to perform a full radiographic examination of both knees was given by 134 floor layers and 120 graphic designers [3]; among these a random sample of 92 and 49 had an MRI of both knees, respectively. Examinations were conducted at two MRI centres over a one-year period (2005–2006) (Figure 1) [4]. Permission was obtained from the Central Danish Region Committee on Biomedical Research Ethics.

### The KOOS

KOOS is an instrument to assess the participants’ opinion about their knee and associated problems. KOOS consists of five subscales:

A.**Pain** (nine items); (1) How often knee pain is experienced. Amount of knee pain, during: (2) twisting/pivoting on the knee; (3) straightening knee fully; (4) bending knee fully; (5) walking on flat surface; (6) going up or down stairs; (7) at night while in bed; (8) sitting or lying; and (9) standing upright.

B.**Other Symptom**s (seven items); (1) Swelling in the knees; (2) Grinding, clicking or any other type of noise when knee moves; (3) Suddenly locking of the knee; Possibility of: (4) straighten the knee; (5) bending the knee; Severity of knee joint stiffness after: (6) wakening in the morning (7) after sitting, lying or resting later in the day.

C.**Function in daily living (ADL)** (17 items); the degree of difficulty experienced while: (1) descending stairs; (2) ascending stairs; (3) rising from sitting; (4) standing; (5) bending to floor/pick up an object; (6) walking on flat surface; (7) getting in/out of car; (8) going shopping; (9) putting on socks/stockings; (10) rising from bed; (11) taking off socks/stockings; (12) lying in bed; (13) getting in/out of bath; (14) sitting; (15) getting on/off toilet; (16) heavy domestic duties; (17) light domestic duties.

D.**Function in sport and recreation (Sport_Rec)** (five items); Degree of difficulty in: (1) squatting; (2) running; (3) jumping; (4) twisting/pivoting on injured knee; and (5) kneeling.

E.**Knee related Quality of life (QOL)** (four items). (1) Awareness of knee problems; (2) modified life style to avoid potentially damaging activities; (3) trouble with lack of confidence in the knee; (4) degree of difficulty with the knee, in general.

Description of the KOOS and the questionnaire can be downloaded from http://www.koos.nu/index.html.

Symptoms during the last week were used. Answers to each item provided a score from 0 (no problems) to 4 (extreme problems). A normalized score (100 indicating no symptoms and 0 indicating extreme symptoms) was calculated for each subscale [10].

### Radiographs

Radiological examinations of both knees were obtained in the standing weight-bearing position with the knee in 20-30^o^ flexion in three views: posterior-anterior, lateral and axial of the patella-femoral (PF) joint space. Radiographs were read and scored on workstations with 2 K screens by one experienced musculoskeletal radiologist unaware of any medical history of knee disorders among all participants and blinded to occupational affiliation. The radiographic scoring comprised assessment of the medial and lateral joint spaces of both the TF and PF compartments using a modified Ahlbäck scale (grade 0–6) of joint space narrowing (JSN) and subchondral bone attrition. The following grades of JSN were defined: grade 0=normal; grade 1=minimal but definite narrowing (25% JSN); 2=moderate narrowing (50% JSN); 3=severe narrowing (75% JSN); 4=obliteration of the joint space, “bone on bone but no attrition”; 5=< 5 mm attrition of subchondral bone and 6=≥ 5 mm bone attrition [3]. Knee OA was defined as JSN ≥ 25% in at least one knee and patterns of involvement categorized into medial or lateral TF OA and PF OA.

Symptomatic knee OA was defined as radiographic knee-OA combined with a total KOOS-score, SUM (the sum of five subscales ≤400).

### MRI

MRI was performed by a 1.5 Tesla scanner in 47 subjects at Centre I, and in 94 subjects at Centre II. MRI sequences were identical for both the right and left knees. The following MRI sequences were obtained at centre I: sagittal proton density fat-saturated turbo spin-echo (TR/TE, 3300/15 ms) and sagittal and coronal T2-weighted (4000/86 ms); coronal T1-weighted (608/20 ms) and axial (3450/15 ms). The section thickness was 4 mm with an intersection gap of 0.4 mm; field of view was 200 × 200 mm and matrix 512 in all sequences. At centre II the MRI sequences included sagittal (TR/TE, 2500/18 ms) and sagittal and coronal T2-weighted (4000/85 ms); coronal T1-weighted (400/13 ms) and axial (2880/17 ms). The section thickness was 4 mm with an intersection gap of 0.4 mm; field of view was 150 × 150 mm and matrix 512 in all sequences.

The medial and lateral menisci of each knee were evaluated separately for abnormalities. In each plane and in each part of a meniscus the observer indicated whether the intra-meniscal MRI-signal had contact with the articular surface indicating a definite meniscal tear. Abnormalities in the MRI-signal intensities (SI) predicting degenerative tears were divided into grades 1–3. For grades 1 and 2 the alterations of the intra-meniscal SI did not extend to an articular surface of the meniscus, whereas at grade 3 was contact to the articular surface. Definite intra-meniscal SI (grade 3) was considered in contact with the surface if it occurred on two contiguous images in either the sagittal or coronal planes or on at least one image in both the sagittal or coronal planes.

A musculoskeletal radiologist with substantial MRI experience evaluated each of the 282 MR examinations. The observer was blinded to any medical history of knee disorders among participants. Due to differences in the MRI appearance from the two centres, blinding of occupational affiliation was incomplete regarding participants from centre I, who were all floor layers. Blinding of occupational affiliation was complete concerning all participants from centre II. The medial and lateral menisci of each knee were evaluated separately for abnormalities. Abnormalities in the MR SI predicting degenerative tears were divided into grades 1–3 [4].

### Data analysis and statistics

Analyses of differences between groups were carried out using multivariate tests for differences between means, and the results were adjusted for age and body mass index (BMI). We use the sum of five subscales to differentiate between symptoms and no symptoms in the present study. In control groups without knee OA KOOS scores were previously found to be in the range 80 and 95 on each subscale [10], and scores for subjects with knee OA < 80. In the present study we provided a total score (SUM) by multiplying 5 subscales by 80 as the boundary between symptoms and no symptoms; and SUM was divided into:>400 (high scores=no symptoms) and ≤400 (low scores=symptoms). Subjects with knee OA combined with a KOOS score ≤400 were defined as ‘symptomatic knee OA’, and subjects with MR-demonstrated meniscal tear combined with KOOS score ≤400 as ‘symptomatic meniscal tear’.

The adjusted odds ratio (OR) with 95% confidence interval (CI) for symptomatic TF OA (JSN ≥ 25%), PF OA (JSN ≥ 25%), and medial meniscal tears (grade 3) was computed in logistic regression models; and independent variables incorporated in the model of adjusted results were age, BMI, knee-straining sports, and earlier knee traumas. Sports activity (i.e. yes/no) was considered as potentially knee straining if it was one of the following: football, handball, badminton, tennis, volleyball, ice hockey, weight lifting, and skiing. Earlier knee traumas (rupture of ligaments, meniscal tears operations, and fractures including the knee joint) were categorized into two groups (i.e. yes/no).

The software package EpiData was used to code data and statistical analyses conducted with SPSS statistics, version 17.0.

## Results

### Characteristics of the study sample

All subjects in the study were males; aged 42–70 years at the time of the examination (mean age 55.6, SD 6.8 years). There was a marked difference in the age distribution and trade seniority between the two study groups. Graphic designers were generally older and had a higher seniority than floor layers. The proportion of participation in knee-straining sports was slightly higher among graphic designers than floor layers. BMI’s of the two groups were comparable (Table 1).

**Table 1:** Characteristics of the study population

	**Floor layers**		**Graphic designers**	
	**(n=134)**		**(n=120)**	
**Age**, years *(mean, SD)*	52.6	6.9	57.9	5.9
**Duration of employment**, years *(mean, SD)*	29.2	10.2	35.6	8.6
**BMI**, kg/m^2^*(mean, SD)*	26.4	3.8	26.0	3.9
**Knee complaints during past 12 months** (yes %)	70	52.2	46	38.3
Knee-straining sports*(yes: n, %)	71	53.0	80	66.7
**Knee osteoarthritis**				
Tibio-femoral, TF OA *(grade 1–3), (n %)*	22	16.4	15	12.5
Patellofemoral, PF OA *(grade 1–3), (n %)*	12	9.0	21	17.5
KOOS:				
*Pain (mean, SD)*	79.98	19.9	89.40	14.3
*Symptom (mean, SD)*	81.28	17.2	89.14	12.3
*ADL (mean, SD)*	82.22	18.9	88.91	16.0
*Sport_re (mean, SD)*	69.51	28.3	78.15	25.9
*QOL (mean, SD)*	67.82	26.6	77.94	23.5
**Meniscal tears**	(n=85)**		(n=40)**	
Medial (*n, %)*	56	65.9	19	47.5
Lateral (*n, %)*	12	14.1	8	20.0

*Defined as football, handball, badminton, tennis, volleyball, ice hockey, and weight lifting.

**Missing: seven floor layers and nine graphic designers, who did not answer the KOOS questionnaire.

### Symptomatic knee disorders measured by KOOS

**Figure 2:**
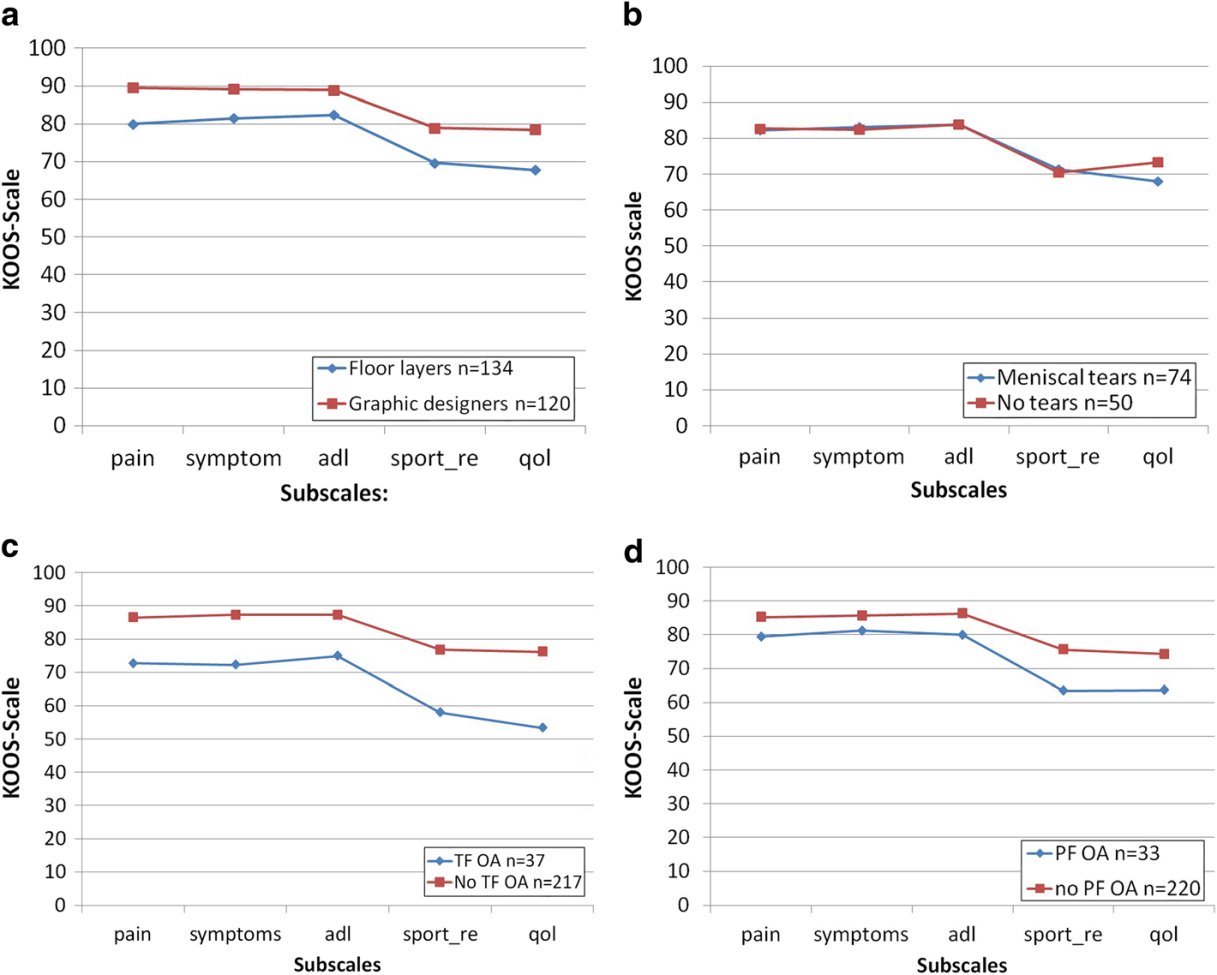
**a. Symptomatic knee disorders measured by Knee injury and Outcome Score (KOOS).** Floor layers and graphic designers. **b**. Symptomatic knee disorders measured by Knee injury and Outcome Score (KOOS). Medial meniscal tears measured by MRI. **c**. Symptomatic knee disorders measured by Knee injury and Outcome Score (KOOS). Tibio-femoral knee osteoarthritis (TF OA). **d**. Symptomatic knee disorders measured by Knee injury and Outcome Score (KOOS). Patello-femoral knee osteoarthritis (PF OA).

Floor layers scored significantly lower on all KOOS subscales compared to graphic designers. There were significantly lower scores on KOOS subscales for radiographic TF OA regardless of trade (Table 2; and Figure 2).

**Table 2:** Group average (mean) for measures of Knee injury and Outcome Score (KOOS) scale (0–100) for pain, symptoms, function in daily living (ADL), sport and recreation (Sport_re), and quality of life (QOL)

**KOOS subscales**	**Pain**		**Symptom**		**ADL**		**Sport_re**		**QOL**	
	**mean**	*P*-value*	**mean**	*P*-value*	**mean**	*P*-value*	**mean**	*P*-value*	**mean**	*P*-value*
Floor layers (n=134)	79.67	< 0.001	81.28	< 0.001	82.22	< 0.001	69.28	0.007	67.57	0.002
Graphic designers (n=120)	89.44		89.57		89.23		78.43		78.53	
Tibio-femoral knee OA**										
yes (n=216)	72.69	< 0.001	71.83	< 0.001	75.08	< 0.001	56.81	< 0.001	53.65	<0.001
no (n=37)	86.19		87.41		87.26		76.40		75.92	
Patello-femoral knee OA**										
yes (n=220)	78.82	0.009	80.69	0.009	79.32	0.001	62.34	0.007	62.50	0.013
no (n=33)	85.03		85.80		86.40		75.21		74.19	
MRI-detected meniscal lesion in one or both knees#										
yes (n=74)	82.64	0.220	83.83	0.280	84.35	0.029	72.36	0.073	69.01	0.039
no (n=50)	81.62		81.76		83.29		69.26		72.34	

* Computed by multivariate analyses and adjusted for covariates (age, BMI, sports, and traumas).

** One missing.

# Missing 17: seven floor layers and nine graphic designers did not answer the KOOS questionnaire, and one did not respond on all the questions in the primary questionnaire.

Subjects with radiographic knee OA showed no differences in scores on KOOS subscales when comparing floor layers and graphic designers (*P* = 0.47).

Symptomatic TF OA (radiographic OA combined with SUM ≤400) was found in eight (6.7%) graphic designers compared to 16 (11.9%) floor layers corresponding to OR 2.6; 95% CI 0.99–6.9 (adjusted for age, BMI, sports, and traumas).

Symptomatic medial meniscal tears (MR-detected medial meniscal tear combine with SUM ≤400) were found in seven (17.1%) graphic designers compared to 26 (31.3%) floor layers with OR=2.04, 95% CI 0.77–5.5 (adjusted for age, BMI, sports, and traumas) (Table 3).

**Table 3:** Occurrence of symptomatic* knee disorders

**Symptomatic:**	**OR**	**95% CI**
Tibio-femoral knee OA	2.60	(0.99–6.9)
(floor layers, n=134, graphic designers, n=120)		
Patello-femoral knee OA (yes/no)	1.35	(0.44–4.1)
(floor layers, n=134, graphic designers, n=120)		
Medial meniscal lesion (yes/no)**	2.04	(0.77–5.5)
(floor layers, n=85, graphic designers, n=40)		

Floor layers compared to graphic designers.

*Symptomatic is defined as KOOS-score ≤ 400 combined with radiographic detected knee osteoarthritis or MRI-detected medial meniscal tear.

** 17 missing (did not answer/or did not answer all questions in questionnaires).

## Discussion

The study showed that floor layers compared to graphic designers had an increased risk of symptomatic TF OA defined by a combination of radiographic signs of OA and knee symptoms measured by KOOS-scores. Previous studies have shown an increased risk of radiographic knee OA in subjects with a high amount of kneeling work activities in their occupations [5,6,11-13]. Radiological changes are most relevant when they also lead to clinical symptoms that may cause difficulties such as working disability and eventually retirement. Floor layers had an increased occurrence of symptomatic knee OA which may be due to the generally increased risk of knee OA in the trade, but it is also possible that floor layers with knee OA have more symptoms in their knees caused by their kneeling work.

In previous population studies from the U.S. and the Netherlands, the prevalence of symptomatic knee OA was shown to be 5.9% for men aged ≥45 years (Framingham study) [14] and 10.1% for men aged 55–64 years (Rotterdam study) [15].

By comparison, in the current study, symptomatic knee OA was found among 11.9% of floor layers, and 6.7% of graphic designers, aged 35–70 years. Prevalence of knee OA for graphic designers in the present study was lower than that in the Netherlands study of the general population but corresponded to the prevalence of symptomatic knee OA among the general male population of same age group in the U.S. [14,15]. The differences may be explained by the definition of ‘symptomatic knee OA’ different age groups, and BMI.

This study showed an increased risk for symptomatic meniscal tears among floor layers compared to graphic designers, measured by a combination of MRI-detected meniscal tears and KOOS-SUM score ≤400 (not significant). Previously we have shown that MRI-demonstrated meniscal tears (with or without symptoms) increased in floor layers compared to graphic designers [4]. Englund et al. showed a 35% prevalence of meniscal tears or destruction in a study population with a mean age of 62 years; 61% of the subjects had no knee pain [16]. This corresponded to an overall prevalence of 14% of symptomatic meniscal tears and compares to the 17% prevalence of symptomatic meniscal tears among the control group of graphic designers in the present study.

Previous studies showed no definite correlation between complaints of knee pain and radiological findings of knee OA although there were more people with knee pain among those with demonstrated severe radiographic knee OA [9]. The present study showed a significant difference in KOOS score for subjects with TF OA compared to those without. This indicates a correlation between symptoms measured by KOOS and knee OA at group level in this population, and is consistent with earlier findings [10].

KOOS-data have been used in several previous studies. KOOS has high test-retest reproducibility [10].

In control groups without knee OA KOOS-scores have been found in the range 80–95. Scores for subjects with knee OA have been found to be <80, the value we used to differentiate between symptoms/no symptoms on subscales in this study (i.e. SUM of subscales was 5 x 80=400).

KOOS-score combined with radiographic or MRI examination gives a more specific description and may qualify the image of symptomatic knee disorders in epidemiological studies.

The strengths of this study are that the floor layers had worked many years in the occupation and had experienced a substantial amount of kneeling work, and that the study included both radiographic, MRI, and self-reported data.

One limitation of the study was the low participation rate which may lead to underestimation of the real risk. There was a high response rate to the questionnaire but only 62% of the floor layers and 52% of the graphic designers participated in the radiographic examination. Results could therefore be biased if the decisions to participate were differentially influenced by previous or current knee complaints. Analysis showed a predominance of graphic designers with self-reported knee complaints and knee traumas among participants in the radiographic study compared to the questionnaire participants (15% of the graphic designers had self-reported knee complaints in the questionnaire study compared to 38% in the radiographic study). However such a selection bias would typically also result in an underestimation of a real risk.

Size of sample for the MRI was chosen as a trade off between statistical power and economic/human resources. As indicated by confidence intervals provided in the tables we could identify relative risks in the range of two or more which we believe is acceptable.

Selection of workers towards different occupations depending on their health status may be inevitable in occupations with high physical demands, and a healthy worker selection may also have influenced results either in terms of primary selection of more healthy workers into the trade or by longer survival in the trade of the most healthy workers. Such a selection bias (healthy worker effect) would typically result in an underestimation of the investigated association.

The control group of graphic designers was significantly older than the floor layers. Since radiological knee OA increases substantially with age this may have had a significant impact on the outcome. Thus, overall there may be an underestimation of the real risk of developing knee OA.

Numbers of years employed in the trade is relevant for overall exposure among floor layers to knee-straining activities. However, it is irrelevant for graphic designers, as they are not exposed to knee-straining work. Therefore differences in seniority between the groups are irrelevant in relation to the overall results.

Previous studies have shown an increased risk of developing knee OA in older age associated with previous severe knee traumas, and specific sports activities. We therefore controlled for these confounders in the present study. The study showed an increased occurrence of symptomatic knee OA in a group of floor layers, who had a substantial amount of kneeling work positions compared to a group without kneeling work positions. Prevention would be appropriate to reduce the proportion of kneeling postures e.g. by working with tools used from a standing working position.

## Conclusion

Floor layers had an increased occurrence of symptomatic knee OA and meniscal tears compared to graphic designers. The KOOS-scores were significantly lower among subjects with knee OA but not in relation to meniscal tears. KOOS-score may qualify the measurement of symptomatic knee OA in epidemiological studies.

## Abbreviations

KOOS: Knee injury and Osteoarthritis Outcome Score; Knee OA: Knee osteoarthritis; TF OA: Tibio-femoral osteoarthritis; PF OA: Patello-femoral osteoarthritis; OR: Odds ratio; CI: Confidence intervals; JSN: Joint space narrowing; MRI: Magnetic Resonance Imaging; 2 K: Reference resolution of the screen (2 K is 2048 x 156 pixels); ADL: Function in daily living; QOL: Quality of life; TR: Repetition Time; TE: Echo Time; T1 and T2: Relaxation times; Sport_re: Sport and recreation; SI: Signal intensities; BMI: Body mass index.

## Competing interests

The authors declare that they have no competing interests.

## Authors’ contributions

LK, corresponding author has the primary responsibility for design, analysis and preparation of the manuscript. SR and JPB were deeply involved in the study design. SR collected data under the supervision of LK and JPB. All authors received the original data and have been deeply involved in the analysis of data and discussion of the manuscript. Each author read and approved the final manuscript.

## Pre-publication history

The pre-publication history for this paper can be accessed here:

http://www.biomedcentral.com/1471-2474/13/188/prepub
